# Varied Clinical Presentations, the Role of Magnetic Resonance Imaging in the Diagnosis, and Successful Management of Cervical Leiomyomas: A Case-Series and Review of Literature

**DOI:** 10.7759/cureus.2653

**Published:** 2018-05-19

**Authors:** Avantika Gupta, Purnima Gupta, Usha Manaktala

**Affiliations:** 1 Obstetrics and Gynecology, Jawaharlal Institute of Postgraduate Medical Education and Research (JIPMER), Puducherry, IND; 2 Obstetrics & Gynecology, Maulana Azad Medical College, New Delhi, IND

**Keywords:** cervical fibroid, mri features, urinary retention, prolapse, leiomyoma

## Abstract

Cervical leiomyomas or fibroids constitute a rare variety of benign pelvic tumors. The symptoms may vary from urinary retention, frequency, dyspareunia to rare clinical presentations such as prolapsed cervical fibroid polyp which may mimic procidentia or even uterine inversion. Preoperative clinical evaluation, radiological imaging, and proper intra-operative delineation of pelvic anatomy can help in their successful management. We are presenting a series of three cases of cervical leiomyomas which presented as a diagnostic challenge but their proper evaluation ultimately led us to manage these cases judiciously. The first case of cervical fibroid polyp mimicked incarcerated procidentia, the second case mimicked pelvic organ prolapse while the third case presented with acute urinary retention. All these cases were evaluated by ultrasound as well as magnetic resonance imaging (MRI) and were managed surgically without any complications. The MRI features of all the cases have been described. One should be aware of the uncommon presentations of cervical fibroid and should consider it in the differential diagnosis of any pelvic mass.

## Introduction

Cervical leiomyomas or fibroids are rare tumors accounting for 0.6%–2% of all the uterine leiomyomas [1]. Cervical fibroid may lead to urinary retention, frequency of micturition, constipation, menstrual abnormalities, dyspareunia or postcoital bleeding [2-3]. Huge cervical fibroid can rarely present as a polypoidal vaginal mass [4-5], can even mimic incarcerated procidentia [6-7] or can masquerade as chronic uterine inversion [8]. It can also cause uterocervical descent as a result of traction [9-10]. Ultrasound and magnetic resonance imaging (MRI) play an important role in the management of patients with cervical fibroid. MRI is very accurate imaging modality to assess leiomyomas [11] in order to detect their number, size, and location. The management of cervical fibroid includes either myomectomy or hysterectomy depending upon patient’s profile. These prove to be a challenge to the surgeon in view of their close proximity to the vital pelvic structures & likelihood to cause surgical complications. We are presenting a series of three interesting cases with different clinical presentations, their diagnostic challenges, and successful management.

## Case presentation

Case 1: Giant cervical fibroid polyp mimicking incarcerated procidentia

**Figure 1:**
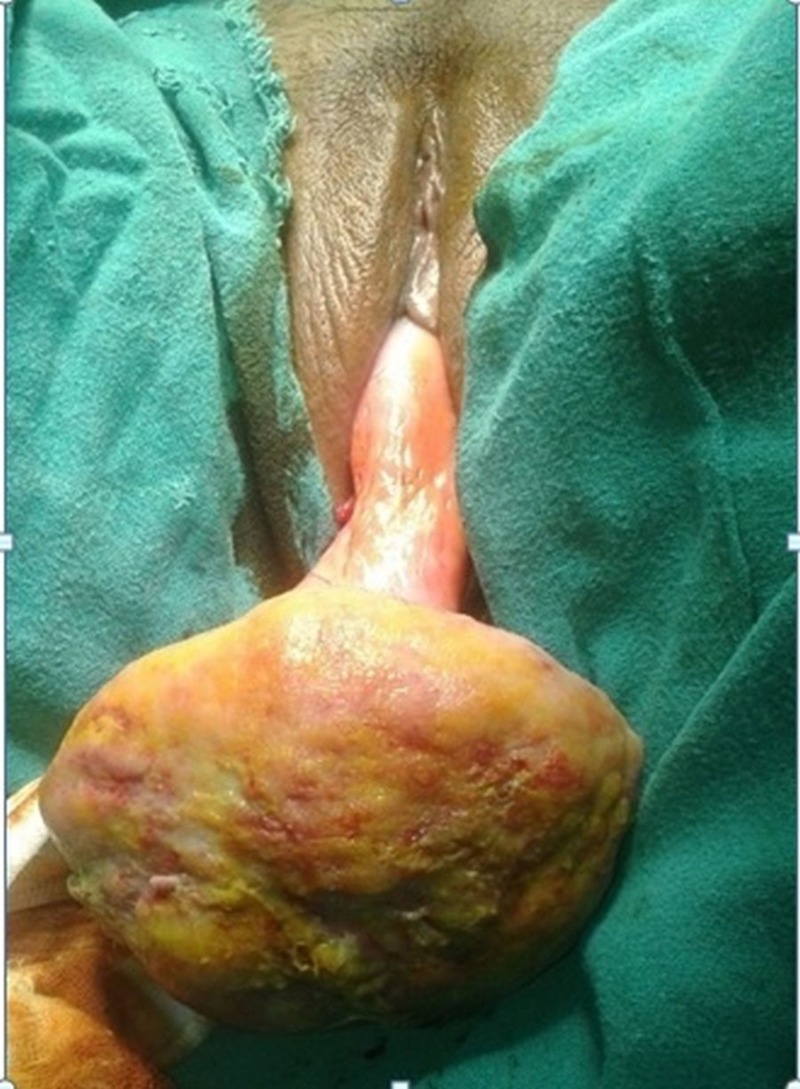
Clinical photograph (Case 1) A huge pedunculated mass dangling from the introitus with ulcerated and necrotic surface

A 47-year-old woman presented with the complaint of huge irreducible mass protruding out of vagina since last two months. She also had complaint of heavy menstrual bleeding and dyspareunia since last one year. General physical examination, systemic examination and per abdomen examination was unremarkable. On local examination, a 13 cm x 13 cm x 8 cm solid fleshy pedunculated mass was seen dangling from the vaginal introitus (Figure 1).

**Figure 2:**
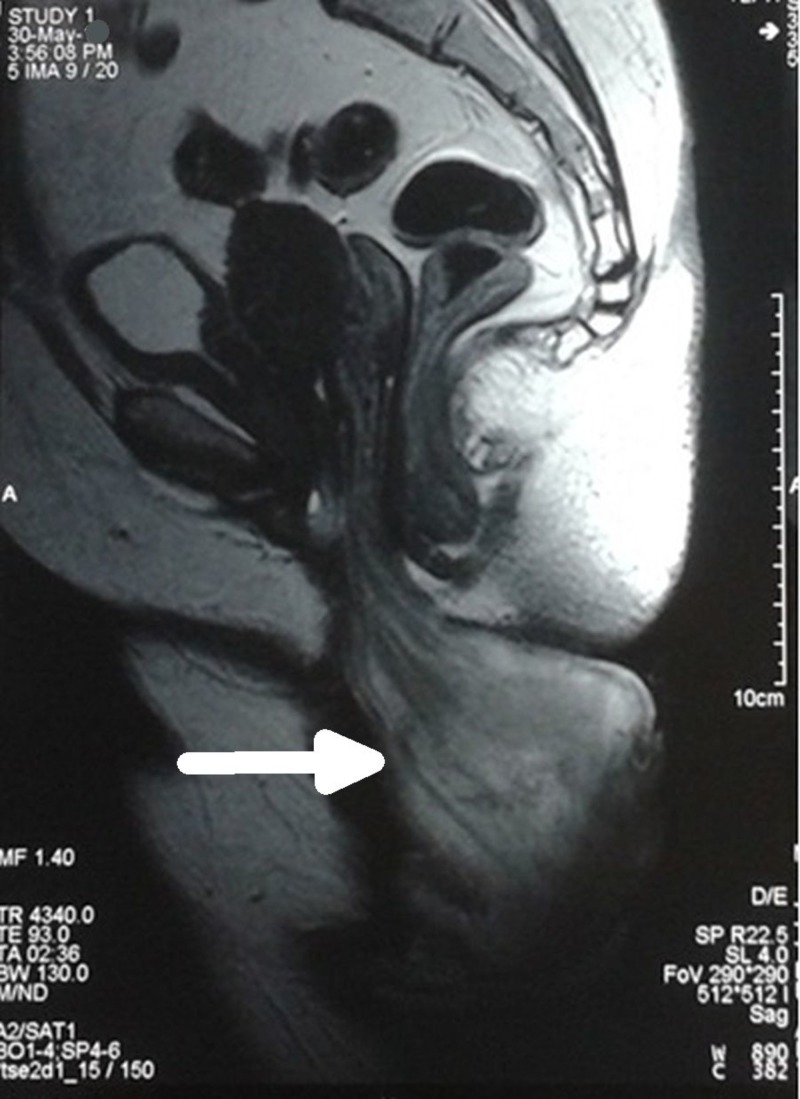
MRI image (Case 1) T2 weighted MRI image of the mass (sagittal view) showing prolapsed mass arising from the cervix and hanging outside vagina (white arrow)

The surface was irregular, friable and necrotic. It had an offensive odor owing to large epithelial tissue slough. The surrounding vaginal walls were hypertrophied & inflamed. On per vaginum examination, pedicle was felt but exact origin could not be ascertained. Bimanual examination was restricted due to mass and neither cervix was felt separately nor uterus was made out. Therefore the exact origin of the mass couldn’t be recognized and the presumptive differential diagnosis of incarcerated procidentia or chronic inversion of uterus or degenerated fibroid polyp was made. Transabdominal ultrasound revealed a normal-sized uterus inside pelvis with a huge protruding vaginal mass. The cervix could not be appreciated separately on ultrasound. MRI pelvis helped us in making the diagnosis of prolapsed cervical fibroid polyp clearly. It showed uterus which was normally placed inside the pelvis & a huge mass measuring 13 cm x 8 cm lying outside introitus originating from cervix (Figure 2).

**Figure 3:**
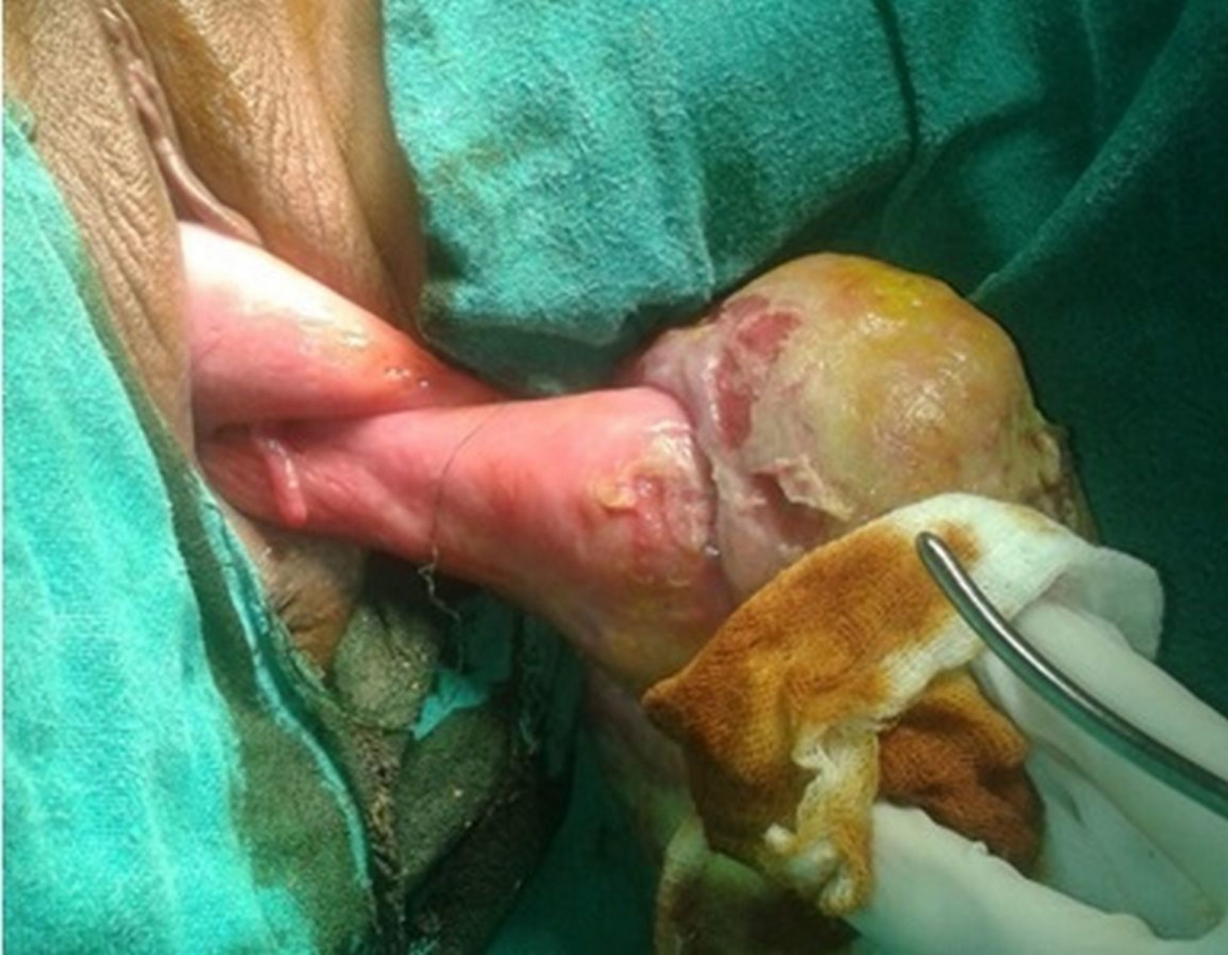
Clinical photograph 2 (Case 1) The pedicle of the cervical fibroid polyp arising from the posterior lip, hypertrophied vaginal walls surrounding both the lips of cervix along with a small endocervical mucosal polyp seen at external os

The patient was managed with parenteral broad spectrum antibiotics, regular dressings of the mass with glycerine-acroflavin solution along with saline irrigation before surgery. Pre-operative biopsy from the surface of the mass revealed only chronic inflammation along with necrosis at the surface without any malignant changes. Actual anatomy of the mass and its relationship with surrounding structures was delineated when the patient was examined under anesthesia in operation theatre. After retracting the mass, we could visualize the external os and a huge fibroid polyp was seen originating from the posterior lip of the cervix surrounded by hypertrophic vaginal walls (Figure 3).

After amputation of the cervical fibroid polyp, normal anatomy of the cervix, surrounding vagina and pelvic floor was identified. We proceeded with hysterectomy as patient also had abnormal uterine bleeding and desired hysterectomy. Intra-operative blood loss was average and there were no intra-operative or post-operative complications. Histopathology of the specimen also confirmed the diagnosis of leiomyoma with degenerative changes at the surface.

Case 2: Cervical fibroid polyp mimicking pelvic organ prolapse

**Figure 4:**
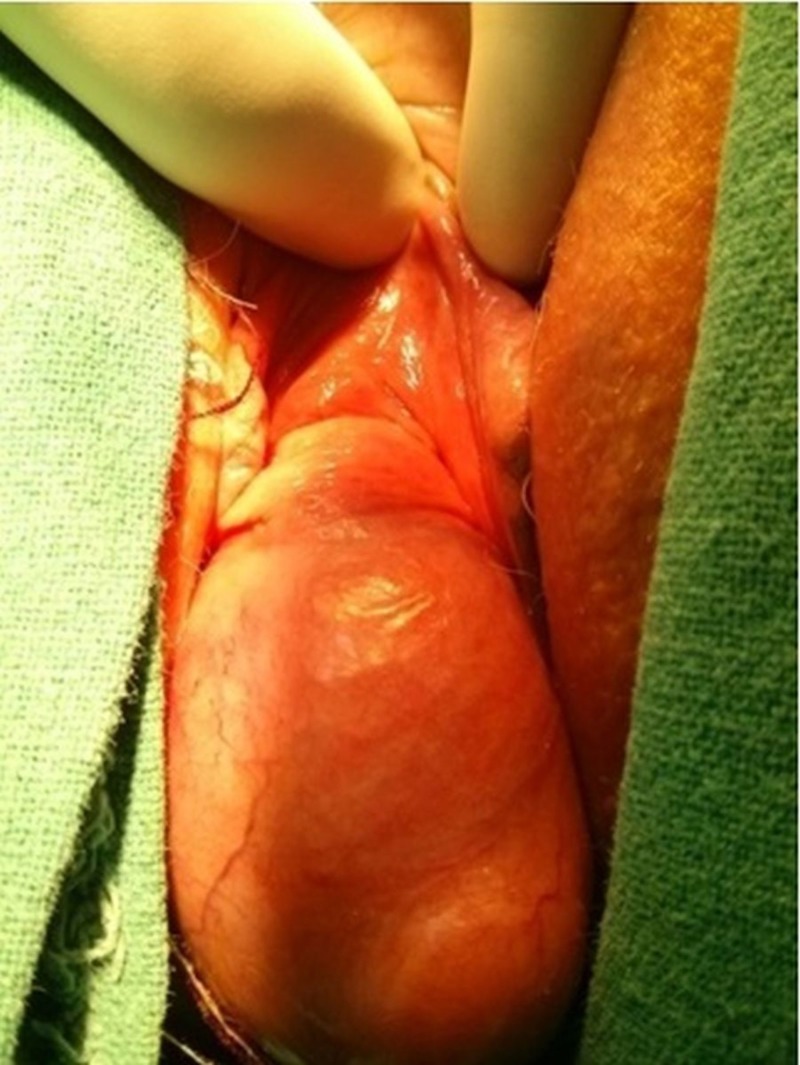
Clinical photograph (Case 2) Anterior cervical fibroid polyp mimicking pelvic organ prolpase

A 45-year-old multiparous lady presented with the complaint of a mass coming out of vagina since last three years which was gradually increasing in size over the time. The mass was reducible and the protrusion of the mass was usually preceded by prolonged standing, sitting in squatting position and straining. She also had complaints of lower abdominal pain, dragging sensation, and dyspareunia since last two years. The symptoms mimicked the clinical presentation of pelvic organ prolapse. General physical examination, systemic examination and per abdomen examination was unremarkable. On inspection, a 5 cm × 5 cm mass was seen with overlying stretched vaginal wall (Figure 4) and mimicked cystocele.

**Figure 5:**
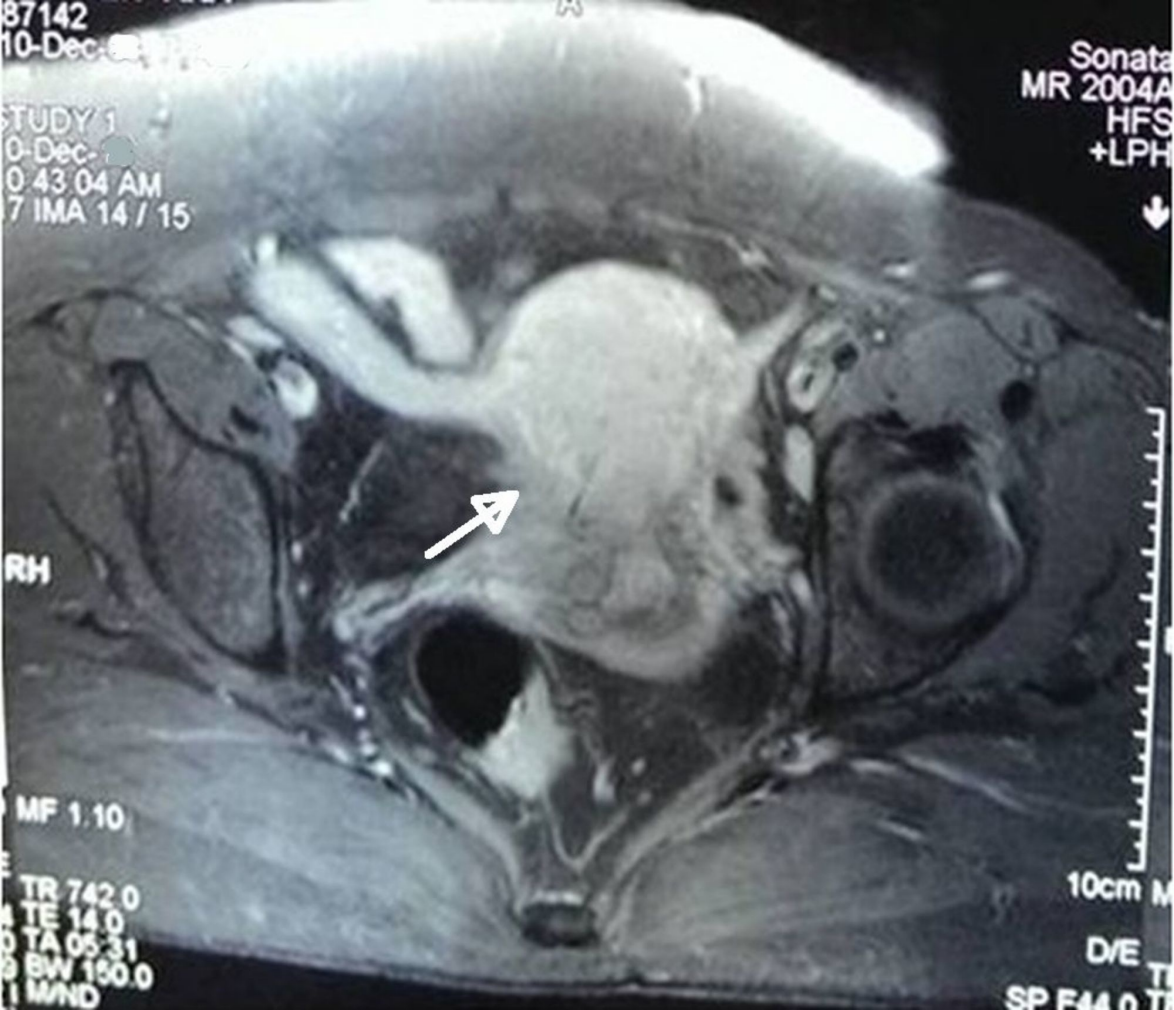
MRI image (Case 2) T2 weighted MRI image showing normally placed uterus and a fibroid polyp arising from the anterior lip of cervix (white arrow)

However, on per vaginum examination, the mass was felt arising from anterior lip of cervix and didn’t transmit cough impulse. On bimanual examination, the normal- size uterus was felt separately from the mass. On further examination, no cystocele/ rectocele/ utero-cervical descent were found. Transvaginal ultrasound revealed a similar sized mass arising from the cervix along with a normally placed uterus. MRI pelvis revealed a 5 cm × 5 cm sized cervical leiomyoma with whorled appearance, arising from anterior lip of cervix (Figure 5).

Patient underwent successful vaginal myomectomy without any intra-operative and postoperative complications. Histopathology of the resected specimen confirmed leiomyoma without any secondary changes.

Case 3: Huge cervical fibroid presenting with acute urinary retention

**Figure 6:**
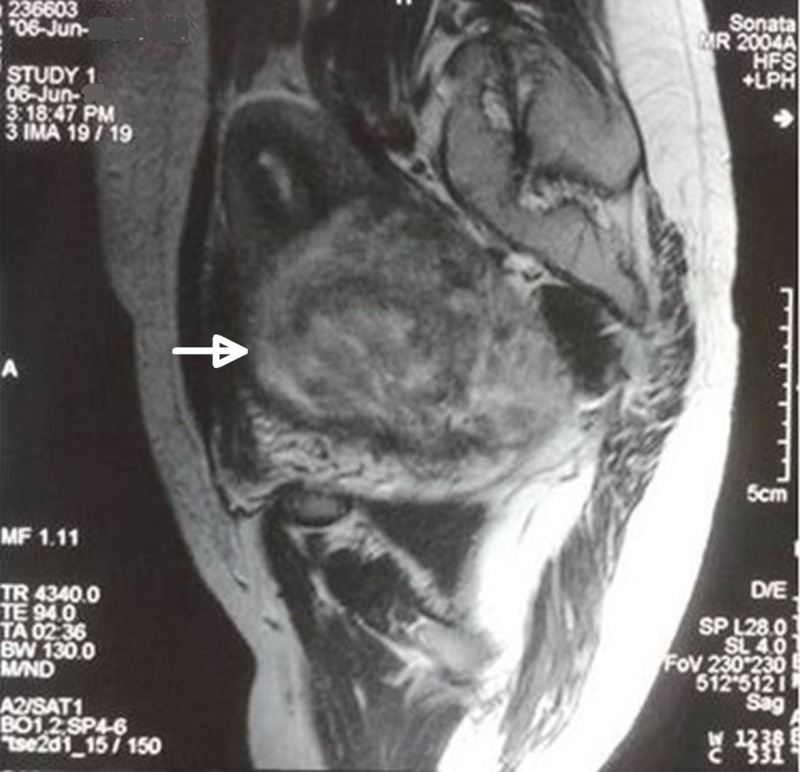
MRI image (Case 3) T2 weighted MRI image (sagittal view) showing a huge cervical fibroid with whorled appearance displacing the uterus anteriorly and upwards (white arrow)

A 30-year-old nulliparous lady presented to emergency room with the complaint of inability to pass urine for last one day along with lower abdominal pain. She also had complaints of excessive bleeding during menses and dyspareunia for the last one year. On clinical examination, the vital parameters were stable. She had mild pallor. Systemic examination was unremarkable. On per abdomen examination, a huge mass was felt reaching upto the umbilicus. Patient was catheterized and bimanual examination was done which revealed a huge mass sized 15 cm x 15 cm, firm in consistency with irregular surface arising from the cervix and occupying the whole pelvis. Ultrasound revealed a same-sized pelvic mass arising from the lower body of uterus and cervix (which was not visualized separately). MRI pelvis revealed a mass of 15 cm × 15 cm size with typical whorled appearance which was arising from cervix and lower uterine body. The uterus was normal sized and was placed just above the cervical fibroid giving it a typical “Lantern of St Paul’s dome” appearance (Figure 6).

MRI also showed mild bilateral hydroureters and hydronephrosis. Her kidney function test and urine analysis were unremarkable. Urine culture didn’t show any growth. Pre-operatively, her hemoglobin was optimized to 12 gm/dl. Considering the complication of obstructive uropathy due to huge cervical fibroid & her parity, we proceeded with abdominal myomectomy. On laparotomy, a large central cervical fibroid measuring 15 cm x 15 cm x 9 cm was seen impacted in the pelvis and displacing the uterus upwards. After careful delineation of the surrounding structures and bladder dissection, successful intra-capsular enucleation of cervical fibroid was done. There were no intra-operative complications and patient stood the surgery well. Histopathology of the mass confirmed the diagnosis of leiomyoma. Her postoperative ultrasound showed resolution of bilateral hydronephrosis.

## Discussion

The paucity of smooth muscles in the cervical stroma makes leiomyomas in the cervix uncommon & account for less than 1% of all the fibroids [1]. Cervical fibroid arises mainly from the supravaginal portion of the cervix. Depending upon their location, cervical fibroids may be classified as anterior, posterior, lateral or central. Anterior fibroid bulges forward & undermines the bladder, leading to urinary frequency or retention [2-3]. Posterior fibroid flattens the pouch of Douglas and compresses rectum against sacrum causing constipation. Central cervical fibroid expands the cervix equally in all directions, pushing the uterus upwards to give the typical “Lantern of St Paul’s dome” appearance.

Cervical fibroid polyp presenting as protruding introital mass can be great masqueraders. A huge cervical fibroid polyp can prolapse outside causing total inversion of cervix and can mimic chronic uterine inversion as reported by Nilgun et al. [8]. In the first case, the prolapsed cervical polyp mimicked incarcerated procidentia as the mass was irreducible and the uterus was not made out separately on bimanual examination. However, the MRI pelvis helped in confirming the diagnosis of prolapsed cervical fibroid polyp without any pelvic organ prolapse. Similar cases of huge prolapsed necrotic cervical fibroid polyp have been reported by Khan et al. in 2011 [6] and Ikechebelu et al. in 2012 [7]. However, in both these cases, MR imaging was not done. Successful surgical management was done in both the cases. In the second case of the present study also, cervical fibroid polyp presented as an introital mass and mimicked anterior compartment prolapse. However, on bimanual examination a solid polypoidal mass was felt arising from the anterior lip of cervix. MRI pelvis again helped in confirming the diagnosis. The third case presented with acute urinary retention which was relieved after catheterization of bladder followed by definitive surgical management, i.e., myomectomy. Cervical myoma causing acute urinary retention has also been reported in the literature as few case reports [2-3]. The cervical fibroid was huge and deeply impacted in the pelvis to cause urinary retention.

Although ultrasound is the preferred method for initial evaluation, MRI is the most accurate method for preoperative localization of leiomyomata & surgical planning for myomectomy [12]. It helps in providing imaging planes that are not available on transabdominal or transvaginal ultrasound, a feature that permits better visualization of the more lateral & posterior area of pelvis. Fibroids appear as sharply marginated areas of low to intermediate signal intensity on T1 and T2 weighted MRI scans [13].

Vaginal myomectomy can be performed in pedunculated cervical fibroid polyp where there is adequate vaginal access and mobility of the mass owing to long pedicle, as present in the second case. In the first case also, the fibroid polyp could be easily removed before proceeding to hysterectomy owing to its long pedicle. Central cervical fibroid is difficult to operate because uterine vessels are so elevated as to run parallel to ovarian vessels forming a vascular leash close to the uterus. Surgical difficulties associated with cervical myomas are poor access to operative field, difficulty in suturing the repairs, increased blood loss as well as injury to the neighboring structures due to distorted pelvic anatomy. After considering the location of myoma, myomectomy or hysterectomy can be performed safely by developing a uniform strategy. The tumor may be impacted in the pelvis displacing the ureters and overhangs the vaginal vault so much that this can’t be reached until the myoma is dislocated upwards or removed by myomectomy. A similar operative difficulty was faced in the third case but was managed successfully by intra-capsular enucleation of the fibroid. Intra-capsular enucleation of the cervical fibroid is the best approach to prevent injury to the bladder and ureters.

## Conclusions

Cervical fibroids are rare and their management can be quite challenging. They present with varied manifestations posing difficulties in diagnosis and management. Good anatomical and clinical judgment is critical to their successful management. Thorough pre-operative evaluation and anticipation of operative challenges lead to judicious treatment.
